# One-Step Ultrasonic Preparation of Stable Bovine Serum Albumin-Perovskite for Fluorescence Analysis of L-Ascorbic Acid and Alkaline Phosphatase

**DOI:** 10.3390/bios13080770

**Published:** 2023-07-28

**Authors:** Lei Deng, Feng Huang, Aomei Zhang, Tingting Wang, Minghui Yang, Xiaoqing Li, Xiang Chen

**Affiliations:** 1 Hunan Provincial Key Laboratory of Micro & Nano Materials Interface Science, College of Chemistry and Chemical Engineering, Central South University, Changsha 410083, China; 192301004@csu.edu.cn (L.D.); huangfengchn@163.com (F.H.); 212311020@csu.edu.cn (A.Z.); 212311008@csu.edu.cn (T.W.); 2 Furong Labratory, Changsha 410083, China; 3 National Engineering Research Center of Personalized Diagnostic and Therapeutic Technology, Changsha 410083, China; 4 The Department of Dermatology, Xiangya Hospital, Central South University, Changsha 410008, China

**Keywords:** perovskite, fluorescence sensing, alkaline phosphatase, L-ascorbic acid

## Abstract

Halide lead perovskite has attracted increased attention due to its excellent optical properties. However, the poor stability of the halide lead perovskite nanocrystals has been a major obstacle to their application in biosensing. Here, we proposed a method to synthesize CsPbBr_3_/BSA NCs perovskite using bovine serum albumin (BSA) as a zwitterion ligand. Then, a fluorescent sensor for alkaline phosphatase determination based on CsPbBr_3_/BSA NCs was successfully built via the interaction of L-ascorbic acid (AA) with BSA on the perovskite surface. Under optimal conditions, the sensor showed a linear concentration range from 50 to 500 μM with a detection limit of 28 μM (signal-to-noise ratio of 3) for AA, and demonstrated a linear concentration range from 40 to 500 U/L with a detection limit of 15.5 U/L (signal-to-noise ratio of 3) for alkaline phosphatase (ALP). In addition, the proposed fluorescent biosensor exhibited good selectivity and recovery in the determination of ALP in human serum. This strategy offers an innovative way for enhancing the water stability of lead halide perovskite and promoting their application in biosensing areas.

## 1. Introduction

Halide lead perovskites have emerged as an appealing nanomaterial in optical and electronic applications owning to the high tolerance of defect, broad light absorption and high photoluminescence quantum yield [1]. The narrow and symmetrical fluorescence emission with a full width at half maximum (FWHM) of 12–42 nm and the large Stokes shift of perovskites made it a desired material for fluorescent bio-applications [2,3]. However, the ionic perovskite is easily destroyed in highly polarized water which is a key obstacle for perovskite biosensor. Many efforts have been made in improving the stability of perovskite in aqueous solution, including crystal structure engineering, defect engineering and surface encapsulation [4,5]. Among the above, surface modification is the most effective method, which enhances the stability of perovskite by the exchange of the ligand or coating materials while preserving the fluorescent structure of perovskite.

Polymer molecular chains were widely used for encapsulating perovskite nanocrystals, such as polystyrene (PS) [6], polymethacrylate (PMMA) [7], polyvinylpyrrolidone (PVP) [8] and some hydrophobic polymer [9]. Although hydrophobic polymer-coated perovskites offer a high potential for bioimaging due to their long-term fluorescence emission and water durability, they respond poorly to environmental changes in biosensing. Inorganic nanoparticles with voids, such as metal organic frame (MOF) or mesoporous silicon, have also been used for perovskite protection and have applied in variable ink printing and photoelectrochemical (PEC) biosensing [10,11,12]. When dispersed in an aqueous solution, these voids inorganic nanoparticles struggle to stop water molecules from eroding perovskite, and it was still a challenge to maintaining the optical properties of perovskite by inorganic nanoparticles coating. Nevertheless, some ligands and small biomolecules were reported to connect with perovskite and were successfully applied in biosensing. Shen et al. proposed a PEC biosensor for the determination of dibutyl phthalate (DBP) based on perovskite coated with cetyltrimethylammonium bromide (CTAB). The static interaction of NH_4_^+^ and Br^-^ between CTAB and the surface of perovskite maintains its stability when different charges of aptamer react with the surface CTAB causing PEC changes [13]. Dong et al. developed aflatoxin M1 (AFM1) and carcinoembryonic antigen (CEA) biosensor based on perovskite via a ligand engineering strategy. Oleylamine-OH (OAm-OH) ligands formed a hydrophilic layer on the surface of the perovskite, where the hydrophobic long chain of OAm protects perovskite from water erosion. This kind of fluorescent biosensor based on perovskite with ligands hydroxyl functionalization possesses good water dispersion and stability [14]. These findings point to the possibility of improving perovskite biosensing via a ligand–perovskite surface interaction.

Recently, ligands or small molecules containing sulfur elements have been reported to stabilize perovskite [15,16]. For instance, a sensitive biosensor for the detection of H_2_S was achieved by creating a strong Pb–S bond with perovskite [17,18]. The ligands with sulfur, such as 1-dodecanethiol (DSH) [19], 4-aminobenzene sulfonic acid (SA) [20] and dodecylbenzene sulfonic (DBSA) [21], were found to improve the fluorescence of perovskite in water, indicating that it is of great potential to modify perovskite using ligands containing sulfur. Since bovine serum albumin (BSA) is composed of 585 amino acid residues, it contains numerous disulfide bonds and a free sulphydryl group, which possesses good biocompatibility and ionic affinity and has been widely used in the fabrication of nanoparticles. [22,23]. Given the numerous groups and unique structure, BSA has a great potential for the surface engineering of perovskite nanocrystals and is expected to increase their aqueous solution stability. As far as we known, there was no study on the modification of perovskite surface using BSA has been reported. In this work, we employed BSA as a ligand to create a perovskite solution with stable luminescence in aqueous solution using an ultrasonic technique. The sulfur groups, such as SH or S-S of BSA, can be well combined with lead ions or defects on the surface of perovskite, which improves the aqueous dispersion and stability of perovskite nanoparticles. In addition, the possible electrostatic interaction between AA and BSA might cause the aggregation of CsPbBr_3_/BSA nanocrystals, resulting in fluorescence quenching. Based on this principle, a fluorescent biosensor based on CsPbBr_3_/BSA was effectively developed for ascorbic acid (AA) and alkaline phosphatase (ALP) determination. This method provides a new way for the future modification of perovskite and the application of perovskite for biosensor design.

## 2. Materials and Methods

### 2.1. Materials and Apparatus

Lead bromide (PbBr_2_, 99%), cesium bromide (CsBr, 99%), oleylamine (OAm, 80~90%) and oleic acid (OA, 85%) were purchased from Aladdin Biochemical Technology Co., Ltd. (Shanghai, China). N,N-Dimethylformamide (DMF, ≥99.5%) was purchased from Macklin Biochemical Technology Co., Ltd. (Shanghai, China). Bovine serum albumin (BSA) was purchased from BBI Life Sciences Co., Ltd. (Shanghai, China). Phosphatase, alkaline from bovine intestinal mucosa buffered aqueous solution (ALP, P6774-2KU), L-Ascorbic acid (AA, 99%) and Tris (hydroxymethyl) amino methane hydrochloride (Tris-HCl) were purchased from Sigma-Aldrich. Ascorbic acid 2-phosphate magnesium ester (AAP, 98%) was purchased from Bepharm Science & Technology Co., Ltd. (Shanghai, China). Human serum samples were received from the Xiangya hospital (Changsha, China). All other reagents were of analytical grade and used without further purification. Ultrapure water (18.2 MΩ cm resistivity at 25 °C, Milli-Q) was used in all experiments.

Fluorescence spectra were measured by F-7000 fluorescence spectrometer (Hitachi, Japan). The transmission electron microscopy (TEM) images were obtained using JEOL JEM-2100 F electron microscopy. The micromorphology and energy-dispersive spectroscopy (EDS) were measured by scanning electron microscopy (FE-SEM, Hitachi S-4800). X-ray diffraction (XRD) was conducted on X-ray diffractometer (XRD-7000, Shimadzu, Japan). UV–vis absorption spectra were recorded using a UV-2450 spectrophotometer (Hitachi, Japan). X-ray photoelectron spectroscopy (XPS) was measured using K-Alpha spectrometer (Thermo Scientific, Waltham, MA, USA). The ultrasonic treatment was conducted using an ultrasonic homogenizer (KQ-500DE, Suzhou, China).

### 2.2. Preparation of CsPbBr_3_/BSA

Water stable fluorescent CsPbBr_3_/BSA nanocrystals was synthesized with ultrasonic treatment from Cs_4_PbBr_6_. Typically, 0.638 g CsBr (3 mmol) and 0.367 g PbBr_2_ (1 mmol) were mixed with 30 mL of DMF stirred continuously for 2 h until a milky-white solution was formed. Then, 1 mL of OA was added quickly and 1 mL of OAm was added slowly, before the upper layer solution was removed after continuous stirring for 1 h. Next, 20 mL of toluene was added, the Cs-rich Cs_4_PbBr_6_ precipitation was centrifuged at 8000 rpm and was then freeze-dried for further use. A total of 10 mg of BSA was dissolved in 10 mL Milli-Q water, mixed with 20 mg of prepared Cs_4_PbBr_6_ and then an ultrasonic treatment was applied for 4 h (40 kHz, 500 W), before it was centrifuged at 4000 rpm to remove large particles. Finally, the CsPbBr_3_/BSA supernatant was prepared and stored at 4 °C.

### 2.3. CsPbBr_3_/BSA-Based Fluorescence Probe for AA Determination

A total of 100 µL of Tris-HCl (10 mM, pH = 7.4) containing different concentrations of AA (0, 0.05, 0.1, 0.2, 0.3, 0.5, 1, 5, 10 mM) was mixed with 100 µL of CsPbBr_3_/BSA solution and oscillated for 5 min at room temperature. The fluorescence intensity of these mixture was measured with F-7000 fluorescence spectrophotometer with the excitation wavelength at 400 nm (EX slit = 5 nm, EM slit = 5 nm, PMT voltage = 700 V).

### 2.4. CsPbBr_3_/BSA-Based Fluorescence Probe for ALP Determination

A total of 80 µL of Tris-HCl (10 mM, pH = 8) containing 5 mM AAP was mixed with 20 µL of ALP with various concentrations (40, 50, 100, 200, 300, 400, 500 U/L) and incubated for 30 min at 37 °C. A total of 20 µL of the above mixture was added into 80 µL of Tris-HCl (10 mM, pH = 7.4) and mixed with 100 µL of CsPbBr_3_/BSA solution, before it was oscillated for 5 min at room temperature. The fluorescence intensity of these mixtures was recorded using an F-7000 fluorescence spectrophotometer with the excitation at 400 nm (EX slit = 10 nm, EM slit = 5 nm, PMT voltage = 700 V).

### 2.5. Human Serum Sample Analysis

The fluorescent biosensor determination of ALP in human serum samples was evaluated using the spike-recycling method. Fresh human serum samples of healthy people were obtained from the Xiangya hospital (Changsha, China). A total of 80 µL of Tris-HCl (10 mM, pH = 8) containing 5 mM AAP was mixed with 20 µL of human serum samples (diluted 10 times) spiked with different concentrations of ALP and incubated for 30 min at 37 °C before the fluorescence measurement (Excitation = 400 nm, EX slit = 10 nm, EM slit = 5 nm, PMT voltage = 700 V).

## 3. Results

**Scheme 1:**
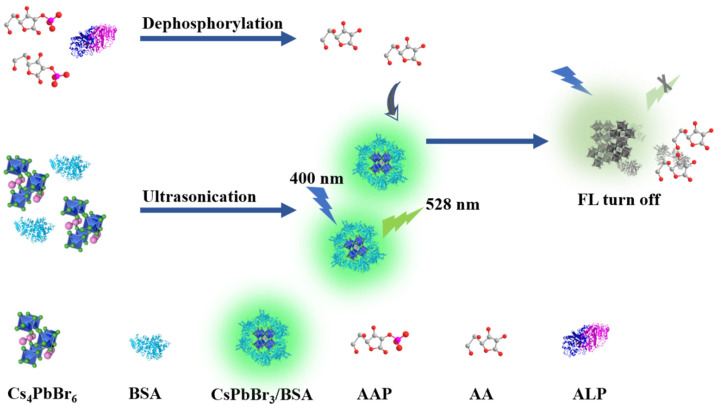
Schematic diagram of fluorescent biosensor based on CsPbBr_3_/BSA nanocrystals for determination of AA and ALP.

In this work, the water-stable CsPbBr_3_/BSA perovskite was obtained by the mixing of Cs_4_PbBr_6_ and BSA with ultrasonic treatment. The binding force between the sulfur-containing groups in BSA and the defects or ions on the surface of CsPbBr_3_ perovskite can produce fluorescence stable perovskite in aqueous solution. However, this binding force was prone to break down, owing to the possible stronger electrostatic force of ascorbic acid (AA) with BSA. In the presence of ALP, AA was produced by the dephosphorylation of AAP, which then reacted with the BSA on the surface of CsPbBr_3_/BSA, resulting in the quenching of the fluorescence (Scheme 1).

### 3.1. Characterization of the Synthetic Process of CsPbBr_3_/BSA NCs

**Figure 1:**
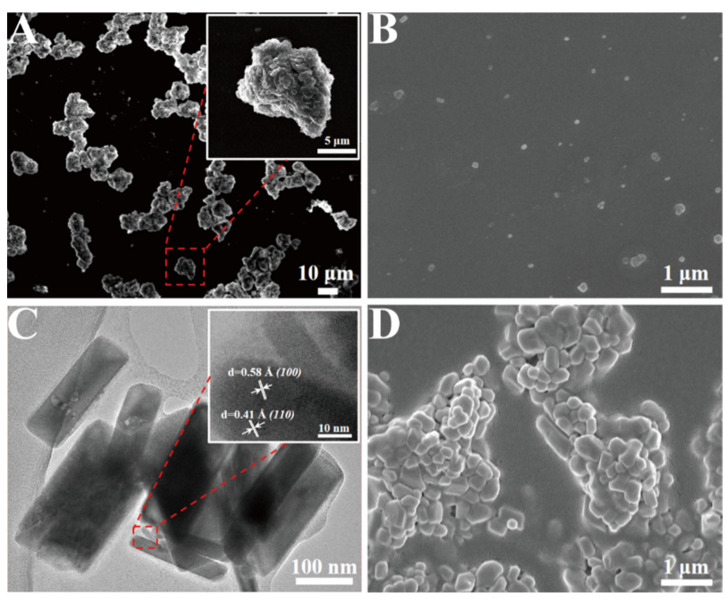
(**A**). SEM image of Cs_4_PbBr_6_; (**B**). SEM image of CsPbBr_3_/BSA.; (**C**). TEM and HRTEM (insert) image of CsPbBr_3_/BSA NCs; (**D**). SEM image of the product of CsPbBr_3_/BSA NCs with AA.

The morphology of Cs_4_PbBr_6_ and CsPbBr_3_/BSA NCs were characterized by SEM and TEM. As shown in Figure 1A, the size of rhombohedral Cs_4_PbBr_6_ was approximately 10 µm with a rough surface, which may be caused by rapid precipitation in DMF when OAm was added [24]. The loose structure of Cs_4_PbBr_6_ will decompose fast in polar solvent, and then be transformed to large bulk CsPbBr_3_, before eventually being exfoliated to CsPbBr_3_ NCs in the aqueous phase with a longtime ultrasonic treatment [25]. The BSA aqueous solution exfoliated fluorescence CsPbBr_3_/BSA nanocrystals with different sizes (Figure 1B), rather than quasi-two-dimensional nanosheets. The reason for this might be attributed to the weak hydrogen bond between the sulfydryl (-SH) and amidogen (-NH_2_) and the strong Pb–S bond which caused different growth rate in different dimensions [26]. As shown in Figure 1C, the lattice spacing were measured to be 0.41 Å and 0.58 Å, which belongs to CsPbBr_3_ lattice planes of (110) and (100). Figure 1D shows the morphology of the product of CsPbBr_3_/BSA and AA, it can be seen that most of the nanoparticles were agglomerated together, and this may be the reason why AA caused fluorescence quenching of CsPbBr_3_/BSA NCs. And the energy-dispersive spectroscopy (EDS) mapping (TEM) of CsPbBr_3_/BSA NCs was shown in Figure S1, which confirmed the uniform distribution of the Cs, Pb, Br, S and C elements. These results indicate that the BSA-coated CsPbBr_3_/BSA NCs were successfully synthesized.

**Figure 2:**
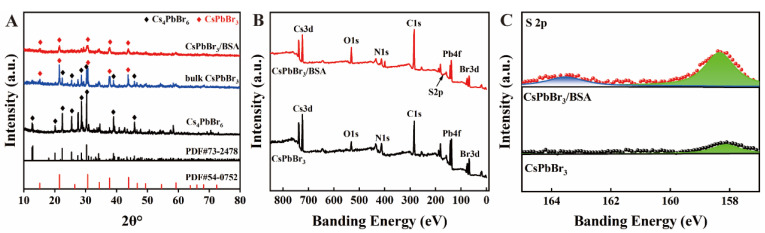
(**A**). XRD patterns of synthesized Cs_4_PbBr_6_, bulk CsPbBr_3_ and CsPbBr_3_/BSA NCs; (**B**). XPS survey spectra of CsPbBr_3_ and CsPbBr_3_/BSA NCs; (**C**). High-resolution XPS scans for S 2p.

X-ray diffraction (XRD) analysis was also used for the characterization of the synthesis process. As shown in Figure 2A, the pristine Cs_4_PbBr_6_ diffraction peaks at 12.9°, 18.1°, 22.4°, 25.4°, 28.6°, 30.3° and 39.0° correspond to the lattice planes of (110), (202), (300), (024), (214), (223) and (324) (refer to JCPDS NO. 73-2478). When dispersed in water, Cs_4_PbBr_6_ quickly transformed into the large CsPbBr_3_ cubic crystal. The XRD pattern of bulk CsPbBr_3_ showed major characteristic cubic CsPbBr_3_ structure (JCPDS NO. 54-0752) lattice planes of (100), (110), (200), (211) and (220) with diffraction peaks at 15.2°, 21.6°, 30.6°, 37.8° and 43.4°, respectively. Several characteristic Cs_4_PbBr_6_ diffraction peaks still existed in the XRD pattern of CsPbBr_3_, which suggests that the nonluminous product of Cs_4_PbBr_6_ in water was the mixture of CsPbBr_3_ and Cs_4_PbBr_6_. The weak diffraction peaks of CsPbBr_3_/BSA NCs indicate that the crystalline shape of the nanoparticles obtained through ultrasonic peeling is not regular, which may be due to the Brownian motion of ions in aqueous solution or the coating caused by BSA. CsPbBr_3_/BSA NCs were scanned and calibrated at high resolution using C1s as the reference peak for X-ray photoelectron spectroscopy (XPS) testing; the testing results are shown in Figure 2B,C. The S 2p peak at 163.5 eV is larger than the common Pb–S bond characteristic peak at 162 eV [15], while there was no peak at 163.5 eV of S 2p in the sample of CsPbBr_3_, and the peak at 158.5 eV belongs to Cs 4p_3/2_, indicating that the sulfur element of BSA does not form PbS with the lead element on the surface of perovskite and that the BSA and CsPbBr_3_ NCs may be bound by a force stronger than the hydrogen bond and weaker than the Pb–S bond. The results of high-resolution XPS scans for elements of C, O, Br and Pb were exhibited in Figure S2.

### 3.2. Optical Properties of CsPbBr_3_/BSA NCs

**Figure 3:**
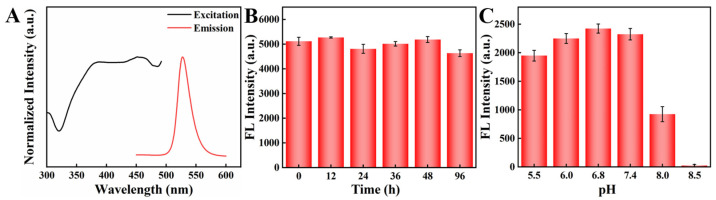
(**A**). Fluorescence spectra of CsPbBr_3_/BSA NCs; (**B**). Fluorescence intensity changes of CsPbBr_3_/BSA NCs in aqueous solution; (**C**). The fluorescence intensity of CsPbBr_3_/BSA NCs corresponding to the value of pH.

The optical properties of CsPbBr_3_/BSA NCs were investigated. As shown in Figure 3A, an emission peak at 528 nm with a FWHM of 25 nm was observed and can be effectively excited from 360 nm to 490 nm, which conforms to typical perovskite nanocrystals material and is suitable for fluorescence sensing. The stability of CsPbBr_3_/BSA NCs in aqueous solution was displayed in Figure 3B, the fluorescence intensity of CsPbBr_3_/BSA NCs hardly change for even 96 h, which may be due to the stabilizing effect of BSA. Additional variables influencing the fluorescence of the solution were also explored, with pH being the most relevant. The fluorescence of perovskite tends to be rapidly quenched especially in alkaline surroundings, due to the structural transitions of BSA in alkaline conditions (Figure 3C). Considering that ALP performs its best catalytic activity under alkaline conditions, the buffer solution we used for subsequent experiments was pH = 7.4.

**Figure 4:**
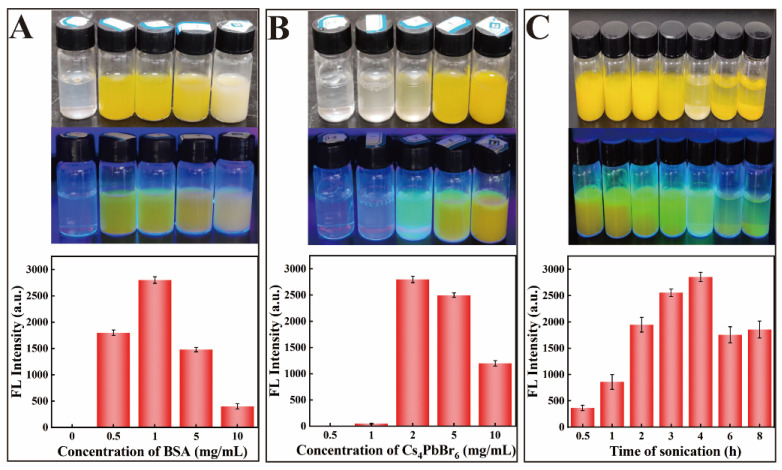
Optimal conditions of CsPbBr_3_/BSA synthesis. (**A**). Concentration of BSA (0, 0.5, 1, 5 and 10 mg/mL); (**B**). Concentration of Cs_4_PbBr_6_ (0.5, 1, 2, 5 and 10 mg/mL); (**C**). Time of ultrasonication (0.5, 1, 2, 3, 4, 6 and 8 h). From up to down, the digital photographs in visible, ultraviolet light and fluorescence intensity (emission at 528 nm), respectively.

The fluorescence intensity of CsPbBr_3_/BSA NCs synthesized through different conditions are illustrated in Figure 4. To establish the appropriate concentration of BSA in the experiment, a series of BSA solutions with varying concentrations (0, 0.5, 1, 5 and 10 mg/mL) were ultrasonically mixed with 2 mg/mL Cs_4_PbBr_6_ for 4 h. According to Figure 4A, the fluorescence intensity was greatest when the concentration of BSA was 1 mg/mL in the aqueous solution. The fluorescence of the solution obtained by ultrasound with excessive concentration of BSA was weakened, which may be due to the high concentration of BSA, and more prone to oxidation and condensation under long-term ultrasonic treatment. Similarly, high Cs_4_PbBr_6_ concentration also led to a decrease in fluorescence intensity of CsPbBr_3_/BSA NCs, which may be due to the large amount of Cs_4_PbBr_6_ at high concentrations, requiring stronger or longer ultrasonic treatment for exfoliation, while Cs_4_PbBr_6_ at small concentrations (smaller than 1 mg/mL) tend to break down during long time ultrasonication. As shown in Figure 4B, the optimal concentration of Cs_4_PbBr_6_ was 2 mg/mL for synthesized CsPbBr_3_/BSA NCs. The optimal time of ultrasonic treatment was 4 h to avoid oxidation induced by extended ultrasound (Figure 4C).

### 3.3. CsPbBr_3_/BSA NCs for Determination of AA

**Figure 5:**
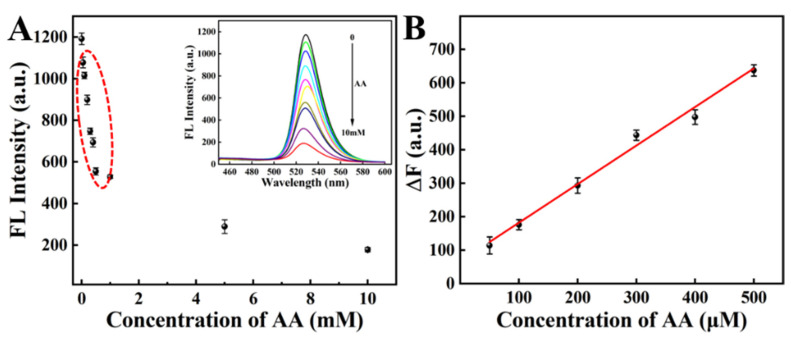
(**A**). The fluorescence intensity of CsPbBr_3_/BSA NCs with different concentrations of AA (Data in red circle is for linear curve).; (**B**). The corresponding linear curve of determination of AA.

L-ascorbic acid (AA), as an essential micronutrient involved in many biological reactions in human body, has been demonstrated as a significant biomarker of many diseases. As shown in Figure 5, the fluorescence intensity at 528 nm of CsPbBr_3_/BSA NCs decreased with the increase of AA. There is a linear relationship between the change of fluorescence intensity and the concentration of AA ranging from 50 to 500 μM. The linear regression equation is Δ*F* = 1.15*c*_(AA)_ + 66.82, the coefficient (*R*^2^) is 0.9904, Δ*F* represents the fluorescence intensity of blank minus the sample’s and the *c*_(AA)_ is the concentration of AA. The limit of detection (LOD) is 28 μM (3*σ*/*S*, where *σ* is the standard deviation of the fluorescence intensity measured by 10 blank samples; *S* is the slope of the linear equation).

The possible mechanism of the AA-induced fluorescence quenching of CsPbBr_3_/BSA NCs was also investigated. We found that some flocculent precipitates were produced in the CsPbBr_3_/BSA NCs solution with the addition of large concentration of AA, as shown in the Figure S3, it can be seen by EDS that the precipitate was uniformly distributed by Pb, Cs, Br and other elements, indicating that it was formed by the condensation of CsPbBr_3_/BSA NCs. In addition, we obtained a CsPbBr_3_ perovskite fluorescence solution without BSA through long-term ultrasonic treatment. As shown in Figure S4, even if 10 mM of AA was added, the fluorescence intensity at 528 nm did not decrease greatly. These results show that the reaction with BSA may be the reason for the decrease in CsPbBr_3_/BSA NCs fluorescence caused by AA, while the mechanism of the reaction needs to be researched further, given there are few studies on AA and BSA reactions to date. In addition, the possible mechanism of AA induced fluorescence quenching of CsPbBr_3_/BSA NCs may be due to the electrostatic interaction between AA and BSA, according to the reported literature [27].

### 3.4. CsPbBr_3_/BSA NCs for Determination of ALP

**Figure 6:**
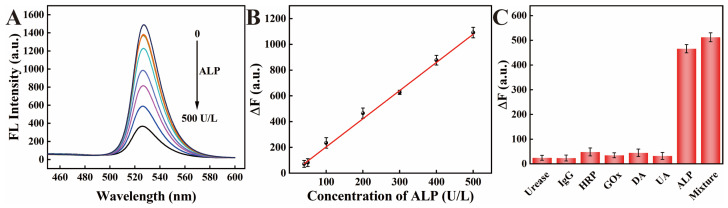
(**A**). The fluorescence intensity of CsPbBr_3_/BSA NCs with different concentrations of ALP (From up to down: 0, 40, 50, 100, 200, 300, 400, 500 U/L of ALP).; (**B**). The corresponding linear curve of determination of ALP.; (**C**). The fluorescence intensity of CsPbBr_3_/BSA NCs with ALP and other interferents.

Alkaline phosphatase (ALP), a common exocrine enzyme in vivo, was a mediator to catalyze the transphosphorylation or hydrolysis of phosphoric acid monoesters in many biological processes, which has been widely reported as biomarker for many diseases. The biosensor in this work realizes the sensitive detection of ALP by quenching the fluorescence at 528 nm of CsPbBr_3_/BSA NCs with different concentrations of AA produced by ALP (Figure 6A). There is a linear relationship between the fluorescence intensity of CsPbBr_3_/BSA NCs and the concentration of ALP, ranging from 40 to 500 U/L (Figure 6B). The linear regression equation is Δ*F* = 2.19*c*_(ALP)_ −14.99, the coefficient (*R*^2^) is 0.9959, Δ*F* represents the fluorescence intensity of blank minus the sample’s and the *c*_(ALP)_ is the concentration of ALP. The limit of detection (LOD) is 15.5 U/L (3*σ*/*S*, where *σ* is the standard deviation of the fluorescence intensity measured by 10 blank samples; *S* is the slope of the linear equation). The proposed fluorescent perovskite biosensor exhibited a suitable linear range covering both normal and abnormally high ALP levels in vivo, as well as a low detection limit for ALP determination.

In order to investigate the specificity of the fluorescent perovskite biosensor, different interference substances were added for analysis, including other proteins in serum (GOx, IgG, HRP, Urease: 10 μg/mL) and some small molecules with a structure similar to AA (DA, UA: 1 mM). As shown in Figure 6C, in the presence of the target ALP (200 U/L), the difference in fluorescence intensity (Δ*F*, represents the fluorescence intensity of blank minus the sample’s) of ALP is much larger than that of other interferents, suggesting that the proposed fluorescence biosensor has good specificity for ALP determination.

### 3.5. Human Serum Sample Analysis

To assess the applicability of the PEC biosensor, the sensor was used to determine ALP in human serum samples using a standard addition method. A fresh human serum sample was received from Xiangya hospital and was diluted 10 times for analysis. Specific amounts of ALP (100, 200 and 300 U/L) were added to the normal people serum, respectively. Each sample was tested three times (*n* = 3). The recovery rates were 96.6%, 108.8% and 101.7% with RSDs of 2.3%, 4.7% and 1.6% (Table 1). The RSDs of all these samples were all less than 5%, indicating that the fluorescent biosensor possesses good accuracy and stability and may be used for analysis of ALP in human serum samples.

Currently, the fluorescent perovskite reported perovskite-based biosensors (Table 2) were almost synthesized using the HI (hot-inject) or the LARP (ligand assisted reprecipitation) method, which require a large amount of organic solvents, such as toluene or *n*-hexane, in the synthesis process, confined to detect polar substances in non-polar solvents or through further complex modification to disperse in an aqueous solution for the determination of biomarkers. In this work, the fluorescent perovskite nanoparticles we used were synthesized in an aqueous solution of BSA subjected to ultrasonic treatment, which avoids the environment pollution caused by the use of volatile organic solvents or the possible injuries caused by high temperature operation. The perovskite-based fluorescent biosensor we proposed realized the detection of ALP in vitro, which has wide application potential in the field of biomarker detection and diagnosis. Compared with other ALP biosensors Table S1), the proposed perovskite biosensor in this work possesses suitable detection range and lower LOD.

## 4. Conclusions

In this work, a strategy was proposed for the green synthesis of fluorescent CsPbBr_3_/BSA NCs with the participation of BSA under ultrasonic treatment, in which BSA played a role in maintaining the stability of the perovskite. The addition of BSA cannot only improve the stability of perovskite but must also provide a possible molecular recognition of ascorbic acid through the electrostatic interaction. The L-ascorbic acid, produced through enzymatic dephosphorylation of AAP by ALP, will combine with BSA on the surface of CsPbBr_3_/BSA NCs, resulting in the quenching of the fluorescence of CsPbBr_3_/BSA NCs solution. The sensitive detection of ALP was successfully realized through this principle. The fluorescent biosensor based on the water-stable CsPbBr_3_/BSA NCs has a wide detection range and low detection limit for ALP detection. However, the mechanisms of the CsPbBr_3_/BSA NCs and AA reaction need to be further studied.

## Figures and Tables

**Table 1:** Determination of ALP in human serum samples (*n* = 3).

Sample	Spiked (U/L)	Found (U/L)	RSD (%)	Recovery (%)
1	---	46.2	4.2	---
2	100	142.8	2.3	96.6
3	200	263.2	4.7	108.8
4	300	351.3	1.6	101.7

**Table 2:** Comparison with several biosensors reported based on perovskites.

	Fluorescent Material	Synthetic Method	Solution	Target Object	Ref.
1	CsPbBr_3_	HI	cyclohexane	H_2_O	[28]
2	CsPbBr_3_	HI	*n*-hexane	H_2_S	[17]
3	CsPbBr_3_	HI	toluene	TPM	[29]
4	CsPbBr_1.5_I_1.5_/MSNs	HI	edible oil	AN	[30]
5	PL-CsPbBr_3_	LARP	water	PSA	[31]
6	CsPbBr_3_/BSA	Ultrasonication	water	ALP	This work

## Data Availability

The data presented in this study are available on request from the corresponding author.

## Supplementary Materials

The following supporting information [32,33,34,35,36] can be downloaded at: https://www.mdpi.com/article/10.3390/bios13080770/s1, Figure S1: EDS digital (TEM) of CsPbBr_3_/BSA NCs; Figure S2. High-resolution XPS scans of CsPbBr_3_ and CsPbBr_3_/BSA NCs; Figure S3. EDS of the precipitate resulting from the reaction of CsPbBr_3_/BSA NCs and AA.; Figure S4. The fluorescence intensity of CsPbBr_3_ NCs (without BSA) with different concentration of AA.; Table S1. Comparison with several reported ALP biosensors.
