# Supplementary material for: One-Step Ultrasonic Preparation of Stable Bovine Serum Albumin-Perovskite for Fluorescence Analysis of L-Ascorbic Acid and Alkaline Phosphatase
Source: Biosensors (Basel). 2023 Jul 28;13(8):770. doi: 10.3390/bios13080770 (PMC10452432; doi:10.3390/bios13080770)
Supplement: Supplementary file 1 [file biosensors-13-00770-s001.zip › biosensors-2503789-supplementary.pdf]

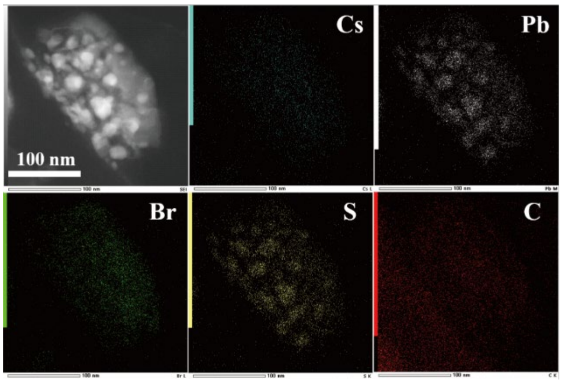

Figure S1. EDS digital (TEM) of $CsPbBr_3$/BSA NCs.

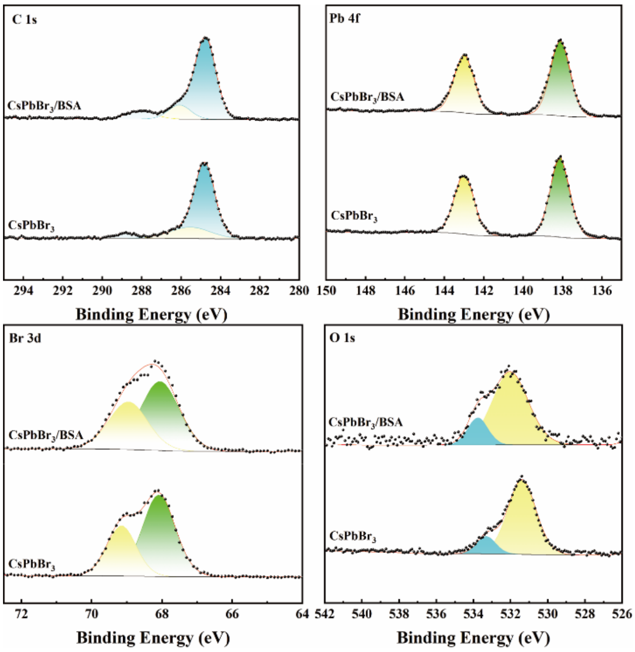

Figure S2. High-resolution XPS scans of $CsPbBr_3$ and $CsPbBr_3$/BSA NCs.

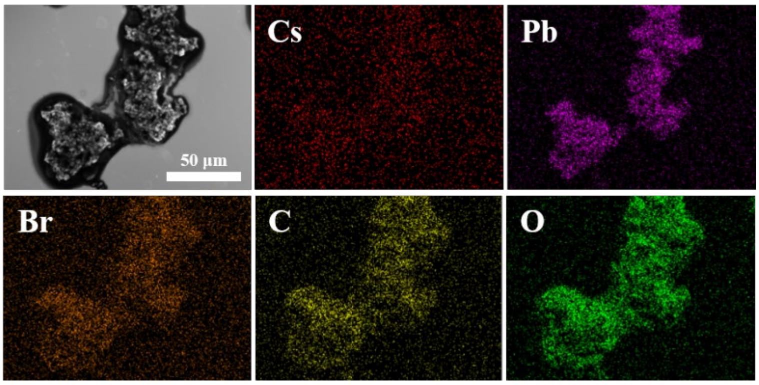

Figure S3. EDS of the precipitate resulting from the reaction of $CsPbBr_3$/BSA NCs and AA.

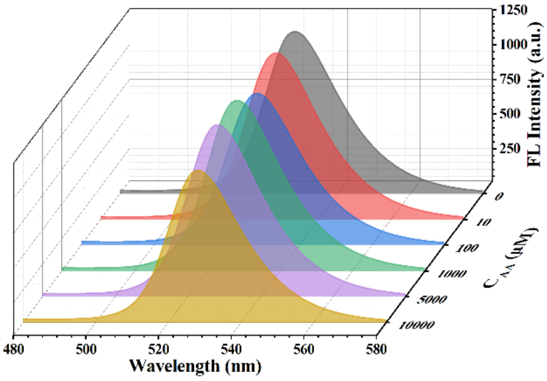

Figure S4. The fluorescence intensity of $CsPbBr_3$ NCs (without BSA) with different concentration of AA.

Table S1. Comparison with several reported ALP biosensors.

Method	Linear range (U/L)	LOD (U/L)	Reference
Fluorescence	1 - 30	0.92	[1]
Chemiluminescence	0 - 400	0.07	[2]
SERS	0.72 - 3	0.01	[3]
DPV	0.1 - 5	0.03	[4]
EIS	100 - 1000	9.1	[5]
Fluorescence	40 - 500	15.5	This work

References

1. Liu, S.G.; Han, L.; Li, N.; Fan, Y.Z.; Yang, Y.Z.; Li, N.B.; Luo, H.Q. A ratiometric fluorescent strategy for alkaline phosphatase activity assay based on g-C3N4/CoOOH nanohybrid via target-triggered competitive redox reaction, Sens. Actuators B: Chem. 283 (2019) 515-523. https://doi.org/https://doi.org/10.1016/j.snb.2018.12.052.
2. Liu, X.T.; Fan, N.N.; Wu, L.J.; Wu, C.C.; Zhou, Y.Q.; Li, P.; Tang, B. Lighting up alkaline phosphatase in drug-induced liver injury using a new chemiluminescence resonance energy transfer nanoprobe, Chem. Commun. 54(88) (2018) 12479-12482. https://doi.org/10.1039/c8cc07211f.
3. Zeng, Y.; Ren, J.Q.; Wang, S.K.; Mai, J.M.; Qu, B.; Zhang, Y.; Shen, A.G.; Hu, J.M. Rapid and reliable detection of alkaline phosphatase by a hot spots amplification strategy based on well-controlled assembly on single nanoparticle, ACS Appl. Mater. Interfaces 9(35) (2017) 29547-29553. https://doi.org/10.1021/acsami.7b09336.
4. Liu, Y.; Xiong, E.; Li, X.; Li, J.; Zhang, X.; Chen, J. Sensitive electrochemical assay of alkaline phosphatase activity based on TdT-mediated hemin/G-quadruplex DNAzyme nanowires for signal amplification, Biosens. Bioelectron. 87 (2017) 970-975. https://doi.org/https://doi.org/10.1016/j.bios.2016.09.069.
5. Mahato, K.; Purohit, B.; Kumar, A.; Chandra, P. Clinically comparable impedimetric immunosensor for serum alkaline phosphatase detection based on electrochemically engineered Au-nano-Dendroids and graphene oxide nanocomposite, Biosens. Bioelectron. 148 (2020) 111815. https://doi.org/https://doi.org/10.1016/j.bios.2019.111815.

Disclaimer/Publisher's Note: The statements, opinions and data contained in all publications are solely those of the individual author(s) and contributor(s) and not of MDPI and/or the editor(s). MDPI and/or the editor(s) disclaim responsibility for any injury to people or property resulting from any ideas, methods, instructions or products referred to in the content.
